# Use of theory in computer-based interventions to reduce alcohol use among adolescents and young adults: a systematic review

**DOI:** 10.1186/s12889-016-3183-x

**Published:** 2016-06-17

**Authors:** Kathleen P. Tebb, Rebecca K. Erenrich, Carolyn Bradner Jasik, Mark S. Berna, James C. Lester, Elizabeth M. Ozer

**Affiliations:** Division of Adolescent and Young Adult Medicine, Department of Pediatrics, University of California, San Francisco, 3333 California Street, Box 0503, San Francisco, CA 94122 USA; Center for Educational Informatics, North Carolina State University, 911 Oval Drive, Engineering Building III (EB3), Room 2402B, Raleigh, NC 27606 USA

**Keywords:** Adolescent, Young adult, Alcohol drinking, Alcohol prevention, Theoretical models, Computer systems, Computer-based interventions, Systematic review

## Abstract

**Background:**

Alcohol use and binge drinking among adolescents and young adults remain frequent causes of preventable injuries, disease, and death, and there has been growing attention to computer-based modes of intervention delivery to prevent/reduce alcohol use. Research suggests that health interventions grounded in established theory are more effective than those with no theoretical basis. The goal of this study was to conduct a literature review of computer-based interventions (CBIs) designed to address alcohol use among adolescents and young adults (aged 12–21 years) and examine the extent to which CBIs use theories of behavior change in their development and evaluations. This study also provides an update on extant CBIs addressing alcohol use among youth and their effectiveness.

**Methods:**

Between November and December of 2014, a literature review of CBIs aimed at preventing or reducing alcohol in PsychINFO, PubMed, and Google Scholar was conducted. The use of theory in each CBI was examined using a modified version of the classification system developed by Painter et al. (Ann Behav Med 35:358–362, 2008).

**Results:**

The search yielded 600 unique articles, 500 were excluded because they did not meet the inclusion criteria. The 100 remaining articles were retained for analyses. Many articles were written about a single intervention; thus, the search revealed a total of 42 unique CBIs. In examining the use of theory, 22 CBIs (52 %) explicitly named one or more theoretical frameworks. Primary theories mentioned were social cognitive theory, transtheoretical model, theory of planned behavior and reasoned action, and health belief model. Less than half (48 %), did not use theory, but mentioned either use of a theoretical construct (such as self-efficacy) or an intervention technique (e.g., manipulating social norms). Only a few articles provided detailed information about how the theory was applied to the CBI; the vast majority included little to no information.

**Conclusions:**

Given the importance of theory in guiding interventions, greater emphasis on the selection and application of theory is needed. The classification system used in this review offers a guiding framework for reporting how theory based principles can be applied to computer based interventions.

## Background

Alcohol use and binge drinking in youth aged 12 to 21 are frequent causes of accidents and injuries, preventable death, disease and psychosocial problems [1]. Though past-month binge drinking^1^ and alcohol use among adolescents and young adults in the United States have declined over the past decade, rates remain high: 23 % report current alcohol use and 14 % binge drinking [2]. Over the past several decades, there have been extensive efforts to address alcohol use among young people. Some interventions have focused on environmental factors (to address youth access) [3] while others have been individual or group level interventions aimed at improving knowledge and attitudes, and reducing alcohol use [4]. These have been primarily face-to-face interventions delivered in structured school or community-based settings.

The application of theory is widely recognized as a crucial component of behavior change interventions. Theories help explain the pathways that lead to or predict behavior and in doing so, provide guidance on how to influence or change behavior. Interventions, that clearly articulate their use of theories, can contribute to a greater understanding of not just what interventions work, but *why* they work. While the interventions targeting alcohol use among youth have resulted in mixed findings, this vast body of work has contributed to the evidence base for what constitutes effective interventions [5, 6]. Interventions that are grounded in established theories of behavior change, and include approaches that address social norms, build self-efficacy and enhance skills to resist pressure to use alcohol, have been found to be more effective than those lacking a theoretical framework [7].

As the field of preventing/reducing alcohol use among adolescents and young adults is evolving, there has been growing attention to the development and use of computer-based modes of intervention delivery [8]. Computer-based interventions (CBIs) have a number of advantages over traditional face-to-face interventions. They are more likely to be implemented with fidelity because they do not rely on the skills, motivation, or time of the facilitator; and they provide a standardized approach to delivering the intervention content [9]. In addition, recent technology innovations enable CBIs to be interactive, provide individually tailored messages and simulate experiences where adolescents can learn and practice skills in convenient and private settings [10, 11]. CBIs also have the potential to be more cost effective than face-to-face interventions [12]. Additionally, computers have become widely accessible and are especially popular among adolescents and young adults [13].

CBIs provide a promising approach to addressing alcohol use among adolescents and young adults. Over the last decade, there have been five literature reviews that have examined the nascent field of digital interventions for alcohol use prevention targeting adolescents and young adults [14–18]. Overall, many of the CBIs have been shown to improve knowledge, attitudes, and reduce alcohol use in the short-term. Three of the five literature reviews examined interventions for college students [14–16]. One review found that CBIs were more effective than no treatment and assessment-only controls, and approximately equivalent to various non-computerized interventions [14]. Another review found that CBIs, when compared to non-CBIs, were more likely to reduce alcohol use [16]. The third review found that CBIs reduced short-term alcohol use compared to assessment-only controls, but not compared to face-to-face interventions [19].

In addition to the reviews focused on alcohol use among young adults, there were two reviews of CBIs targeting younger adolescents. One demonstrated that CBIs delivered in middle or secondary schools effectively reduced alcohol, cannabis and/or tobacco use [17]. The other review was a metanalysis focused on computer games to prevent alcohol and drug use among adolescents and concluded that the games improved knowledge, but it did not find sufficient evidence that these games changed substance use attitudes or behaviors [18]. While these reviews suggest that CBIs have the potential to be efficacious, the mechanisms that contribute to improvements in attitudes and behaviors are not well understood. Use of a theoretical framework helps to explain the mechanisms of change by informing the causal pathways between specific intervention components and behavioral outcomes. Understanding these mechanisms improves our understanding of how and why a particular intervention works.

There has been little attention as to how theoretical frameworks have informed the development of CBIs focused on alcohol use among adolescents and young adults. Only two of the five aforementioned literature reviews covering CBIs for alcohol use in youth examined the underlying theoretical basis of the CBIs [17, 18]. In both of these reviews, the names of the theory and/or specific theoretical constructs were mentioned; however, there was little examination of how the theories were applied to the CBIs. In addition to the reviews focused specifically on adolescent and young adult substance use, there was an additional systematic review that examined the relationship between the use of theory and the effect sizes of internet-based interventions. This study found that extensive use of theory was associated with greater increases in the effect size of behavioral outcomes [20]. They also found that interventions that utilized multiple techniques to change behavior change tended to have larger effect sizes compared to those using fewer techniques. This review builds on prior work demonstrating that health interventions grounded in established theory are more effective than those with no theoretical basis [7, 21–25]. However, this review did not exclusively focus on alcohol use or adolescents specifically. It is therefore important to build upon this knowledge base and focus on the application of theory in CBIs to address adolescent/young adult alcohol use.

The primary goal of this study is to conduct a review of how theory is integrated into CBIs that target alcohol use among adolescents and young adults. Specifically, this study examines which CBIs are guided by a theoretical framework, the extent to which theory is applied in the CBIs and what if any measures associated with the theoretical framework are included in the CBI’s evaluation. A secondary goal is to provide an update of CBIs addressing alcohol use among youth in order to expand our understanding of their effectiveness.

## Methods

The methods follow the guidelines developed and recommended by the PRISMA group.

### Search strategy

PsychINFO and PubMed (electronic databases) were searched to identify peer-reviewed journal articles on computer-based interventions aimed at preventing or reducing alcohol. The search included previous reviews of CBIs. In addition, Google Scholar was searched to identify additional articles/abstracts that may have been published. The reference lists of all the identified articles were also reviewed. The search, which used both Medical Subject Headings [MeSH] and non-MeSH terms, used the search terms: “alcohol abuse prevention,” “alcohol,” “alcohol drinking/prevention and control,” “computer,” “internet,” “web,” “computer software,” “computer games,” and “intervention.” The search was conducted between November and December of 2014.

### Inclusion and exclusion criteria

To be included in this review, the main component of the intervention was required to be delivered via computer, tablet or smartphone. Interventions could include a video game, computer program, or online module. In addition, the intervention needed to target alcohol use among adolescents and young adults between the ages of 12 to 21 years. While adolescence covers a wide range, we chose this age range because there is general consensus that it has begun by age 12, and we included youth up to age 21 since that is the legal drinking age in the U.S. Studies whose participants’ had a mean age between 12 and 21 years were included even when individual study’s participants’ ages extended outside this age range. Interventions intended to treat a substance use disorder were excluded. Non-English language articles, research protocols, and intervention studies that did not report outcomes were also excluded from analyses.

### Data extraction and synthesis

Once eligible studies were identified, the characteristics of the intervention, the context of the intervention, the population targeted, intervention dosage, study author, year and outcomes were entered into a spreadsheet for analyses. Duplicate articles were deleted and journal articles which discussed the same intervention were grouped together. When there was more than one unique article for any given CBI, the CBI was counted only once. In some cases, a given CBI existed in several editions, was modified, or was applied to a different study population. These variations of the CBI were grouped together.

Painter et al.’s classification system was used to categorize the use of theory in each of the CBIs [22]. Consistent with this system, first a CBI was examined to see if an established, broad theory was mentioned in any of the corresponding articles for a given CBI. If so, the CBI was classified as “mentioned”. Second, articles were reviewed to see if they provided any information about how the CBI used theory to inform the intervention. If any of the articles associated with a given CBI provided any information about the use of theory, the CBI was classified as “applied. For our third category, we used “measured” to classify CBIs if any associated article included at least one specific measure of a construct within the theoretical framework. This third category is a slight departure from Painter’s typology which classifies interventions as “tested” if over half of the constructs in the theory are measured in the evaluation of the intervention. We opted for “measured” because testing theories is a complex process and not a common practice of CBIs. We did not use Painter’s 4th category, “building or creating theory” because this was not applicable for any of these interventions.

For all articles reporting on effects of the intervention on alcohol use, attitudes, or knowledge on an included CBI, the effectiveness of the CBI on these outcomes was also examined.

Two senior health research scientists (a counseling/health psychologist and developmental psychologist), with advanced training in theories of behavior change, oversaw the classifications system and addressed questions about the application of a theory/theoretical constructs. The review was conducted by a trained research associate with a master’s degree in public health. A spread sheet was created that included each classification, a description of how the theory was applied, and a list of relevant constructs that were measured.

## Results

**Fig. 1:**
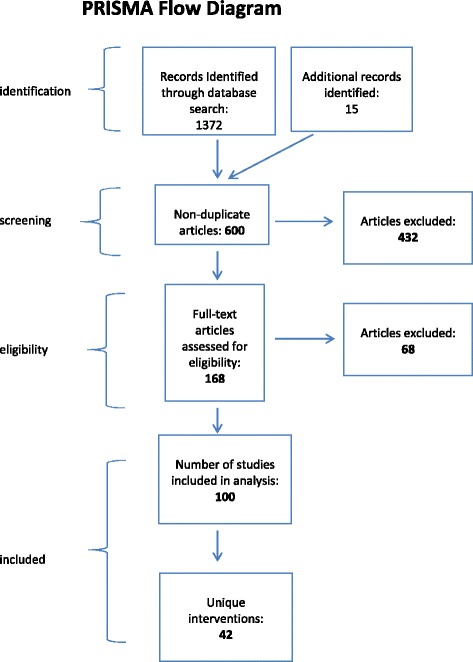
PRISMA flow diagram

The search strategy yielded a total of 600 unique articles published between 1999 and 2014, including 15 articles identified through hand searches and reviews of previous literature reviews. Of these, 500 were excluded because they did not meet the study inclusion criteria. The final sample consisted of 100 articles of 42 unique CBIs. There were more articles than interventions because multiple articles were published on any one CBI intervention. See Fig. 1 for a more full explanation of the articles excluded and yielded during the search process. The list of the 42 interventions and corresponding articles associated with the intervention are provided in Tables 1 and 2. Of the interventions reviewed for this study, 50 % were not included in previous review articles. Of the 42 CBIs in this study, 33 were delivered in school settings and the remaining CBIs were administered in home or in clinic settings.

**Table 1:** Description of theory mention, application, and use by interventions which included an overarching theory

Name of intervention	Related studies	Theories	Mentioned	Applied	Measured constructs	How Theory Applied	Measures of Theoretical Constructs
21 Web Basics [United States]	Neighbors C, 2012	Theory of Planned Behavior	✓	✓	✓	Personalized normative feedback regarding participants’ intended quantity and frequency of alcohol consumption use at 21st birthday. Provides education on alcohol (e.g., the relationship between alcohol consumption and blood alcohol concentration). Asks partcipants to consider alternatives to drinking.	• Drinking intention • Intention to use protective behaviors (e.g., limiting the number of drinks, avoiding drinking games)
	Neighbors C, 2009						
Alcohol 101 [United States]	Barnett NP, 2004	Social Cognitive Theory	✓	✓	✓	Personalized normative feedback about participants’ drinking patterns and perceptions of peer drinking. Includes elements of motivational interviewing (including information intended to enhance risk perception). Informational content, and harm reduction suggestions.	• Attitudes towards alcohol • Motivation to change drinking, assessed with “Readiness Ladder” • Normative and self-ideal discrepancy
	Barnett NP, 2007						
	Carey KB, 2009	Theory of Reasoned Action Transtheoretical model					
	Carey KB, 2010						
	Carey KB, 2011						
	Donahue B, 2004						
	Lao-Barraco C, 2008						
	Mastroleo NR, 2011						
	Murphy JG, 2010						
	Reis J, 2000						
	Sharmer L, 2001						
AlcoholEdu [United States]	Croom K, 2009	Expectancy theory	✓	✓	✓	Presents “ideas of self-efficacy as related to safe and responsible drinking.” Challenges postive expectancies related to the effects of alcohol use on behavior, mood and cognition. Media literacy and knowledge of adverse effects of drinking is linked to social norms theory. A segment of normative feedback built on motivational interviewing techiniques.	• Expectancies of alcohol use: positive and negative • Perceived drinking norms
	Hustad JTP, 2010						
	Lovecchio CP, 2010	Social Cognitive Theory					
	Paschall MJ, 2011						
	Paschall MJ,, 2011	Social norms theory					
	Paschall MJ, 2014						
	Wyatt TM, 2013						
	Nygaard P, 2012						
	Wall AF, 2006						
	Wall AF, 20071						
Climate Schools: Alcohol Module/Alcohol and The CLIMATE Schools Combined [Australia]	Newton NC, 2009	Social Influence Approach, derived from social learning theory	✓	✓	✓	Discussion of alcohol and drug refusal skills alcohol use norms among 14–15-year-olds, decision-making about whether to consume alcohol and the purpose of getting drunk discussed, differing views on the consumption of alcohol.	• Alcohol knowledge • Alcohol expectancies
	Newton NC, 2009						
	Newton NC, 2010						
	Newton NC, 2011						
	Newton NC, 2012						
	Teeson MN, 2014						
	Vogl L, 2009						
College Alc [United States]	Bersamin M, 2007	Problem Behavior Theory	✓	✓	✓	Personalized feedback on how users’ drinking and attitudes towards drinking compare to their peers’. Posting of written assignments and journal entries on a public bulletin board encouraged. Passages about social norms designed to help students clarify their attitudes toward alcohol use and gain a better understanding of peer attitudes. Users encouraged to consider the expectancies they hold regarding alcohol use and how those expectancies influence their behavior.	• Alcohol expectancies (positive and negative) • Alcohol-related knowledge • Alcohol-related attitudes • Intentions to minimize alcohol-related harm • Normative alcohol beliefs
	Paschal MJ, 2006						
		Theory of Planned Behavior					
Check Your Drinking [Canada]	Cunningham JA, 2012	Social Norms Theory	✓	✓	✓	Personalized normative feed back (comparing the participants’ drinking to others of a similar age, sex, and country of origin in the general population or [in the university edition] college student population. Assessment of the severity of the participants’ drinking concerns.	• Perceptions of peer drinking
	Doumas DM, 2008						
	Doumas DM, 2009						
eCHECKUP TO GO (eCHUG) [United States]	Alfonso J, 2013	Expectancy theory	✓	✓	✓	Personalized normative feedback assesses the user’s alcohol use and expectations of alcohol use and provides feedback comparing user’s use to typical college students’ use the extent of the negative consequences the student attributes to her or his alcohol use. Motivational interviewing/ motivational enhancement principles mentioned, application unclear.	• Readiness to change • Motivation to change • Positive alcohol expectancies • Positive beliefs about alcohol use
	Doumas DM, 2009						
	Doumas DM, 2014	Social norms theory					
	Murphy JG, 2010						
	Walters ST, 2007						
	Walters ST, 2009						
	Wodarski JS, 2012						
Lifeskills Training CD-ROM [United States]	Williams C, 2005	Social Learning Theory	✓	✓	✓	The basis of this CBI, the LifeSkills Training program, [60] was developed based on Social Learning Theory. The intervention teaches social, self-management and drug resistance skills. Sessions on building self-esteem; goal setting; decision making; myths and misconceptions about tobacco, alcohol, and marijuana; literacy; anxiety management; communication and social skills; and assertiveness training.	• Life-skills knowledge (e.g., communication skills, assertiveness, refusal skills) • Peer and adult normative expectations regarding smoking, drinking, and drug use • Pro-drug attitudes
		Problem Behavior Theory					
		Self-derogation Theory					
		Peer cluster theories					
Michigan Prevention and Alcohol Safety for Students (M-PASS) [United States]	Barretto AI, 2011	Health Belief Model	✓	✓	✓	Information that relates alcohol consequences to users’ personal values provided. Personalized feedback provided based on a self-efficacy survey and users’ perceptions of alcohol norms. Section on alcohol use myths and facts corrects confusions and reinforces accurate information. Students make choices based on scenarios where they may be tempted or presurred to drink. Users select benefits of and barriers to drinking less or not drinking at all and are presented with a benefits/barriers scorecard. Users set alcohol- or value-related goals and strategies to reach goals, and learn to monitor progress.	• Tolerance of drinking and drive/drinking • Reasons to drink • Use of strategies to avoid high-risk drinking • Motivations for drinking and not drinking alcohol. **Stages of change:** • For high-risk drinkers, the 12-item Readiness to Change Questionnaire • For low-risk drinkers, a single-item about anticipated alcohol use in 6 months
	Bingham C, 2010						
	Bingham C, 2011	Theory of Planned Behavior					
		Transtheoretical Model					
		Precaution Adoption Process Model					
PAS (Prevention of alcohol use in students) [Netherlands]	Koning IM, 2009	Theory of planned behavior	✓	✓	✓	Targets the students’ abilities to develop a healthy attitude towards alcohol use, and build refusal skills.	• Adolescents’ self-control • Attitudes towards drinking and parental rules
	Koning IM, 2010						
		Social cognitive theory					
Project Fitness [United States]	Moore MJ, 2012	Behavior-Image Model (which is supported by Prospect Theory)	✓	✓	✓	Messages on the benefits of health behaviors illustrate how health-promoting behaviors promote salient other and self-images, and messages imparting used to show how health risk behaviors interfere with image outcomes and achievement of health promoting habits.	• Alcohol intentions • Alcohol prototype image [perceived similarity to those who drink] • Willingness to be seen as someone who drinks a lot • Behavior coupling [whether alcohol is perceived to interfere with other health behaviors] • Alcohol social norms
Reach Out Central [Australia]	Burns J, 2007	Social cognitive theory	✓	✓		Players navigate a virtual, realistic environment designed to be engaging and appealing to the audience, meet other characters and engage in a variety of social situations. Scenarios allow players to make choices and see the consequence of their choices. To help youth recognize and learn strategies to improve their mood, the player’s in-game mood is affected by activities and how he or she responds to other characters and situations.	[No specific outcomes pertaining to theories]
	Burns J, 2010						
	Shandley K, 2010	Elaboration likelihood model					
RealTeen [United States and Canada]	Schwinn TM, 2010[b]	Social Learning Theory	✓	✓	✓	Lessons on nine topics: goal setting, decision making, coping, self-esteem, assertion, communication, media influences, peer pressure, and drug facts. Players respond to a question related to each topic, and can post their response to a personal diary, a public blog, or a peer “pen-pal.”	• Self-efficacy to make decisions, set goals, refuse drugs, and manage social situations and stress • Perception of the acceptability of using alcohol • Perceptions of alcohol use norms among peers
What Do You Drink [Netherlands]	Voogt CV, 2011	I-change Model (integration of several approaches including Fishbein-Ajzen’s Theory of Reasoned Action, Transtheoretical Model, and Social Learning Theory)	✓	✓	✓	A personalized normative feedback segment, includes screening and feedback tailored to alcohol intake, sex and perceived social norms, including advice about drinking according to national health guidelines, estimates of the the number of standard drinks and calories consumed, and the cost of those drinks in weight gained and money spent. Another segment asks participants to make decisions about how much alcohol they want to drink, provides them with tips for how to resist alcohol in different situations, shows vignettes related to alcohol use, and asks them to determine factors in the scenes that make it hard to resist drinking. Goal setting and action planning elements related to motivational interviewing.	• Positive or negative attitudes towards alcohol use • Self-efficacy • Subjective norms • Alcohol expectancies
	Voogt CV, 2012						
	Voogt CV, 2013						
	Voogt CV, 2014						
	Voogt CV, 2014						
		Social Influence/Social Cognitive Theory					
Your Decisions Count– Alcohol, Tobacco and Other Drugs [United States]	Evers KE, 2012	Transtheoretical model	✓	✓	✓	Feedback given on progress through the stages of change. Advice is given on what behavioral strategies players could employ to continue progressing. Short movies of students giving testimonials about drug use.	• Pros and cons of being drug-free (decisional balance) • Processes of change • Processes of resistance • Self-efficacy • Stage of change (for each substance being targeted)
No name [Asian-American Mother Daughter Intervention] [United States]	Fang L, 2010	Family interaction theory	✓	✓	✓	Extensive exercises to cultivate trust and communication between mother and daughter: a conflict management role play; animations showing how engaging in or avoiding substance use respectively hurts or benefits adolescent girls; body image and mood management exercises; sress management exercises with animated characters illustrating signs of stress; problem solving using the Stop, Options, Decide, Act, and Self-praise metthod; and exercises correcting misperceptions of peer use of substance with graphs and other visuals; and an interactive game emphasizing the importance of praise and assertiveness.	• Level of mother daughter closeness • Maternal monitoring • Mother-daughter communication
	Fang L, 2012						
	Fang L, 2013						
	Fang L, 2014						
No name [Black and hispanic mother-daughter intervention] [United States]	Schinke S, 2011	Family interaction theory	✓	✓	✓	Activities to improve mother-daughter communication, increase parental monitoring and rule enforcement, build daughters’ self-image and self-esteem, create family rituals, and avoid unrealistic expectations on the part of mothers. Exercises to increase the value of time together and to increase family rituals and routines. Lessons designed to enhance self-efficacy were incorporated into the program (with no explanation of how self-efficacy was enhanced).	• Mother-daughter communication • Perceptions of family rules against substance use • Perceptions of parental monitoring of extracurricular activities, whereabouts, and friends • Normative beliefs about peer substance use • Self-efficacy to avoid alcohol, tobacco, and drug use • Daughters’ intentions to smoke, drink, and use drugs as adults
		Social Learning Theory					
		Attachment Theory					
		Deviant behavior proneness theory					
No name [College freshman intervention] [United States]	Lewis MA, 2007a	Social Comparison Theory	✓	✓	✓	Personalized normative feedback providing information regarding personal drinking, perceptions of typical student drinking, and actual typical student drinking norms. Two versions were created: one offering gender-specific feedback and the other offering gender-neutral feedback.	• Revised version of the Collective Self-Esteem Scale, a measure of gender identity **For peers in general and same-gender peers, perceptions of:** • Typical weekly drinking • Typical number of drinks consumed per drinking occasion • Typical drinking occasions per week
	Lewis MA, 2007b						
		Social Impact Theory					
		Social Identity Theory					
No name [E-newsletter intervention] [United States]	Moore MJ, 2005	Extended Parallel Process Model (based on Social Cognitive Theory and the Health Belief Model)	✓	✓	✓	E-mail newsletter includes a question challenging an alcohol-expectancy belief and refuting that expectancy; presented a “realistic” strategy for reducing the risk of binge drinking	• The questionnaire covered “constructs from prominent psychosocial theories associated with alcohol consumption and underpinning the EPPM, including Social Cognitive Theory and Health Belief Model”; results not published for these measures
No name [Laptop ER intervention] [United States]	Gregor MA, 2003	Social Learning Theory	✓	✓		Intervention based on the Alcohol Misuse Prevention Study curriculum, which in turn was based in Social Learning theory. Content designed to increase knowledge about alcohol, increase refusal skills, and decrease intentions to misuse alcohol. Refusal skills taught by having the participant refuse an offer of beer and then receiving feedback about his or her choice	[No specific outcomes pertaining to theories]
	Maio RF, 2005						
No name [Web-based Substance Use Prevention for Adolescent Girls] [United States]	Schinke S, 2009	Family interaction theory	✓	✓	✓	Exercises designed to build rapport, positive communication and respect between parent and child; emphasizing value of listening to each other, spending time together, understanding one another’s personality, negotiating mutually agreeable resolutions to problems, and giving gifts of time, compliments, and personal favors. Includes modules aimed at refusal skills, self-esteem, goal-setting, racism, assertiveness, peer norms around underage drinking, and conflict and stress management.	• Mother-daughter communication skills • Parental monitoring and rule setting • Drug-refusal self-efficacy. • Normative beliefs assessed with relevant items from the American Drug and Alcohol Survey • Measures of depression, problem solving skills and body esteem.
	Schinke S, 2009						
	Schinke S, 2009

**Table 2:** Description of theoretical constructs and techniques mentioned, applied, and tested among interventions which do not include an overarching theory

Name of intervention	Related studies	Theoretical constructs/ techniques	Mentioned	Applied	Measured constructs	How Theory Applied	Measures of Theoretical Constructs
College Drinker's Checkup [United States]	Hester RK, 2012	Motivational interviewing	✓	✓		Uses “an empathic and nonjudgmental tone” and contains two decisional balance exercises relating to the pros and cons of alcohol use	[No specific outcomes pertaining to constructs or techniques]
		Personalized normative feedback	✓	✓		Personalized feedback on users’ quantity and frequency of drinking, estimated peak blood alcohol concentration, and frequency of alcohol-related problems compares to other, same gender students at their school	[No specific outcomes pertaining to constructs or techniques]
Drinkers Assessment and Feedback Tool for College Students (DrAFT-CS) and DRAFT-CS plus moderation skils [United States]	Weaver CC, 2014	Motivational interviewing	✓	✓		Video of an interviewer provides information in an “empathic, nonjudgmental manner”	[No specific outcomes pertaining to constructs or techniques]
		Personalized normative feedback	✓	✓		Personalized feedback on alcohol use behaviors, consequences, and perceived norms	[No specific outcomes pertaining to constructs or techniques]
e-SBINZ [New Zealand]	Kypri K, 2010	Personalized normative feedback	✓	✓		Personalized normative feedback on measures of unhealthy drinking, estimated blood alcohol concentration, estimated costs of user’s drinking. Harm reduction tips and links to treatment	[No specific outcomes pertaining to constructs or techniques]
	Kypri K, 2013						
Head On, for grades 6 through 8 [United States]	Marsch LA, 2007	Manipulating subjective social norms	✓	✓	✓	Addresses tendency to overestimate the percentage of their peers who use drugs/alcohol	• Beliefs about prevalence of substance use among peers and adults
In Focus [United Kingdom]	Gare L, 1999	No theory or construct mentioned				--	--
iHealth Study [United States]	Saitz R, 2007	Personalized normative feedback	✓	✓		Gender-specific personalized normative feedback presenting local drinking frequency and intensity norms, assessment and feedback on alcohol-related consequences	[No specific outcomes pertaining to constructs or techniques]
		Motivational interviewing	✓		✓	--	• Readiness to change
MyStudentBody.com [United States]	Chiauzzi E, 2005	Personalized normative feedback	✓	✓		Uses a “social norm calculator” to compare users’drinking pattern to peers of same gender, racial or ethnic group, fraternity or sorority membership and athletics participation.	[No specific outcomes pertaining to constructs or techniques]
Project Chill [United States]	Walton MA, 2013	Motivational interviewing	✓	✓		Discussion of goals/values, coping with negative mood, and a decisional balance exercise. In role- plays, participants are asked to make a behavioral choice and consider the consequences in relation to their goals	[No specific outcomes pertaining to constructs or techniques]
	Walton MA, 2014						
		Personalized normative feedback	✓	✓		Graphs comparing participants’ use of cannabis and alcohol to norms for age and gender	[No specific outcomes pertaining to constructs or techniques]
		Self-efficacy	✓	✓		Two segments (“You decide: reasons for avoiding using/reasons for using” and “What we covered”) listed self-efficacy (with little explanation of how they support self-efficacy). Role-playing segment activity to build refusal skills	[No specific outcomes pertaining to constructs or techniques]
Refusal Challenges [United States]	Bryson R, 1999	Self-efficacy	✓	✓		Students role-played twelve high risk situations with computer- simulated peers. Teaches progressively more complex social skills.	[No specific outcomes pertaining to constructs or techniques]
SafERteens [United States]	Cunningham RM, 2009	Motivational interviewing	✓	✓		Decisional balance exercise examines costs of remaining the same and the benefits/reasons for change. A “buddy” character summarizes the reasons the player checked to show the connections between behaviors and goals.	[No specific outcomes pertaining to constructs or techniques]
	Cunningham RM, 2012						
		Personalized normative feedback	✓	✓		Personalized feedback reviews survey responses regarding alcohol,fighting, and weapon carrying and compares users’ behaviors to norms for age and sex.	[No specific outcomes pertaining to constructs or techniques]
		Self-efficacy	✓	✓	✓	“Supporting self-efficacy for making changes” is a stated objective of the “Reasons to Stay Away from Alcohol and Fighting” segment	• Self-efficacy for avoiding alcohol
THRIVE (Tertiary Health Research Intervention Via Email) [Australia]	Hallet J, 2009	Personalized normative feedback	✓	✓		Assesses alcohol use behaviors and provides personalized feedback on AUDIT scores, the risks of the user’s level of drinking. Provides information on alcohol and harm reduction tips.	[No specific outcomes pertaining to constructs or techniques]
	Kypri K, 2009						
No name [At-risk university students personalized normative feedback] [United States]	Butler LH, 2009	Personalized normative feedback	✓	✓		Personalized feedback including a comparison to same-gender peers. Review of the participant’s binge drinking. Information on blood alcohol concentration. Description of calories consumed, money spent and time used drinking. Harm reduction strategies. Mental health and alcohol treatment resources	[No specific outcomes pertaining to constructs or techniques]
No name [Blood alcohol concentration feedback] [United States]	Thombs DL, 2007	Personalized normative feedback	✓	✓	✓	Blood alcohol concentration measurement at night. Feedback on students’ nighttime blood alcohol concentration (BAC) the following morning, including normative feedback comparing of users’ readings to the average BAC in their residence hall the previous night.	• Participants’ estimation of fellow dormitory residents’ blood alcohol concentration
No name [E-mailed personalized normative feedback for college students] [United States]	Bryant ZE, 2013	Personalized normative feedback	✓	✓	✓	Personalized feedback on estimated blood alcohol level during typical and peak drinking sessions, negative consequences, weekly mean number of drinks, gender-specific drinking norms, and the amount of time and money devoted to drinking.	• Number of days participants perceived their peers to have drunk alcohol •Amount of alcohol participants perceived their peers to have consumed per drinking occasion
No name [Gender- specific personalized feedback to reduce alcohol use among college Students] [United States]	Neighbors C, 2010	Personalized normative feedback	✓	✓	✓	Assessment of participant’s drinking behavior, perception of college peer drinking, and graphic and text display of other students’ self-reported drinking behavior.	• Perceived gender- nonspecific and gender- specific drinking norms
No name [New Zealand university student intervention] [New Zealand]	Kypri K, 2004	Personalized normative feedback	✓	✓		Personalized feedback summarizing recent alcohol consumption, participants’ alcohol risk status, estimate of participants’ peak blood alcohol concentration over the last month, comparison of participants’ drinking with national and campus norms and drinking guidelines.	[No specific outcomes pertaining to constructs or techniques]
	Kypri K, 2008						
No name [Intervention to reduce alcohol use among hazardous drinking college Students] [United States]	Palfai TP, 2011	Personalized normative feedback	✓	✓		Personalized feedback on same- gender student norms of total alcohol consumption, heavy drinking episodes, and certain alcohol-related consequences; costs and calories associated with alcohol use; and peak blood alcohol levels.	[No specific outcomes pertaining to constructs or techniques]
No name [Primary care intervention for multiple health risk behaviors] [New Zealand]	Kypri K, 2005	Personalized normative feedback	✓	✓		For each of the health behaviors assessed, information on guidelines, social norms for same age and gender, and a description of the advantages of healthy choices in these arenas.	[No specific outcomes pertaining to constructs or techniques]
No name [Swedish electronic screening and brief intervention] [Sweden]	Elkman DS, 2011	Personalized normative feedback	✓	✓		Personalized feedback consisting of a summary of weekly consumption, frequency of heavy episodic drinking, and highest blood alcohol concentration in the last 3 months; comparison of the respondents’ drinking patterns with safe drinking limits; statements describing participants’ alcohol use compared with university peers; and, if applicable, advice on reducing unhealthy consumption	[No specific outcomes pertaining to constructs or techniques]
	McCambridge J, 2012						
	McCambridge J, 2013						
	McCambridge J, 2013						
No name [U.K. college student intervention] [United Kingdom]	Bewick BM, 2008	Personalized normative feedback	✓	✓		Personalized feedback on the health risks of the participant’s level of alcohol consumption, the percentage of peers who reported drinking less alcohol, and information on calculating units of alcohol, health risks of high levels of alcohol consumption, and drinking guidelines	[No specific outcomes pertaining to constructs or techniques]
No name [Web-based intervention to change perceived norms of college student alcohol use and sexual behavior on spring break] [United States]	Patrick ME, 2014	Personalized normative feedback	✓	✓	✓	Personalized feedback on intended sexual behavior and alcohol consumption over spring break, expected consequences of these behaviors, behavioral norms for age and cohort compared to the participant’s perceived norms, participants’ goals for spring break and motivations to limit alcohol use, protective behavioral strategies, and pacts with friends about alcohol use.	• Normative beliefs about underage drinking

### Summary of included studies

Tables 1 and 2 summarize the included studies. The interventions were largely studied exclusively in the United States (30/42). The remaining interventions were studied in Australia (*n* = 3), New Zealand (*n* = 3), the Netherlands (*n* = 2), the United Kingdom (*n* = 2), Sweden (*n* = 1) and in both the United States and Canada (*n* = 1). Study sample sizes ranged widely. Included studies had between 59 and 20,150 participants. The number of study participants was less than 200 in 26 % of included studies, from 200 to 1,000 in 53 % of studies, and over 1,000 in 21 % of studies. Nearly all interventions had at least one study that measured alcohol use as a primary outcome (*n* = 37). Other common primary outcomes included binge drinking (*n* = 17), perceived alcohol norms (*n* = 14), consequences of alcohol use (*n* = 14), alcohol-related attitudes (*n* = 8), and alcohol-related knowledge (*n* = 6).

### Classification of CBIs

Table 1 provides a list of the CBIs (and corresponding articles) and how theory was used according to the classifications of “mentioned”, “applied” or “measured”. In addition, if the theory was applied to the intervention, a brief description of its application is provided. Similarly if it was classified as “measured” the measure of the theoretical construct was also listed. The CBIs in Table 1 all indicated use of a broad theoretical framework. Broad theories specify the relationship between a number of constructs and associated variables that explain or predict behaviors. Broad theories of behavior change take into account a number of complex contextual factors (e.g. social, cultural, economic, etc.) and inter-related sets of constructs that influence behaviors. CBIs that did not mention use of a broad theoretical framework are listed in Table 2. These CBIs typically mentioned use of a specific theoretical construct without reference to a broader theory, or intervention technique. In addition, sometimes a specific construct or intervention technique can be associated with more than one theory. For example, several of these CBIs mentioned that the goal of the intervention was to improve “self-efficacy”, a specific construct that is most often associated with Social Cognitive Theory [26], but is also incorporated within other theories such as the Theory of Reasoned Action [27]. We applied the same classification system to these CBIs with regard to mention, application and measure for the construct and/or techniques. For each CBI listed in Tables 1 and 2, the use of the theory or construct/technique are classified as (1) mentioned, (2) applied, or (3) measured (using at least one of the theoretical constructs).

#### Theory mentioned in CBIs

Half of the CBIs (21) were affiliated with at least one article that explicitly mentioned use of a broad, overarching theoretical framework in the development of the CBI (see Table 1). Eleven of these mentioned drawing from more than one broad theoretical framework. The primary theories mentioned were Social Cognitive Theory [28] and its predecessor Social Learning Theory [26] (*n* = 10); the Theory of Planned Behavior [29] and the Reasoned Action [27] and the Health Belief Model (*n* = 5) [30]; Social Norms Theory (*n* = 4) [31]; and the Transtheoretical Model (sometimes referred to as Stages of Change Theory) (*n* = 3) [32].

The other half of the CBIs did not mention use of a broad/overarching theoretical framework; however, all but one of these mentioned use of a specific theoretical construct and/or evidence-based intervention technique (see Table 2). Of the 20 CBIs that mentioned a specific construct/technique, personalized normative feedback was mentioned in 18 CBIs, followed by motivational interviewing (mentioned in 5 CBIs), self-efficacy (mentioned twice) and manipulating subjective norms (once).

#### Application of theory in CBIs

As noted above, a CBI was classified as “applied” if any one of the associated articles provided some description of how the theory/construct was used in the CBI. Of the 21 CBIs that mentioned use of a broad theory, all provided at least some information about how the theory was applied to the intervention (see Table 1). However, the quality of the description explaining how the theory was applied varied considerably across the CBIs. Tables 1 provides a brief summary of how the articles, associated with each CBI, applied theory. There were a number of articles that provided a strong description of how the theory was applied to the intervention (e.g. Alcohol Edu [33–41], Michigan Prevention and Alcohol Safety for Students [42–44] and a mother-daughter intervention for black and Hispanic girls [45]). Another intervention, the Life Skills Training CD-ROM [46], was derived from an evidence-based comprehensive in-person curriculum with a strong basis in Social Learning/Cognitive Theory. The Life Skills Training CD-ROM, like the original face-to face curriculum, contains a number of modules that articulate the specific linkages between theory and intervention approaches. Other articles described how one or two aspects of the theory were applied to the CBI, but not the overall theoretical pathway that would inform behavior change (e.g. PAS [47, 48] and a emergency department-based laptop intervention [49, 50]) In contrast, the majority of articles lacked sufficient information to understand how theory informed the development of the intervention.

For the CBIs listed that did not mention use of a broad theory (those listed in Table 2), but mentioned using a specific construct or technique, all provided a description of how it was applied in the intervention (see Table 2); however the amount and quality of information provided about the application of the construct/techniques varied considerable across this group of CBIs.

#### Measurement of theoretical constructs

Of the 21 CBIs that mentioned use/application of theory (in Table 1), all but two included at least one measure of a construct associated with the theory. If a CBI mentioned use of a theory, it was more likely to include a measure of specific constructs associated with the theory compared to CBIs that did not mention use of a broad theory. Specifically, of the CBIs, that did not explicitly mention use of a theory, but did include a specific construct, only five included corresponding measures of the theoretical construct (see Table 2). Tables 1 and 2 lists the classification of each CBI and provides a list of the measure(s) associated with the theory, construct or intervention technique.

### Effectiveness of CBIs

The effectiveness of the CBI was also examined. Tables 3 and 4 provides information about the 83 articles associated with an includedCBI that reported study outcomes: the setting, participants, a brief description of the intervention, comparators and the primary outcome measures that were used to evaluate the effectiveness of the CBI. The measures listed in Table 3 and 4 are primary outcome measures and, in many cases, are different from those listed in Tables 1 and 2 which lists the measures of theoretical constructs which were often secondary rather than primary outcomes. For the outcomes listed in Tables 3 and 4, an asterisk denotes statistical significance (at the level of *p* ≤ 0.05) indicating that the intervention showed more favorable results than the comparator (e.g., lower alcohol use or frequency of binge drinking, greater negative expectancies related to alcohol use, etc.) Of the 42 CBIs, all but one [48] demonstrated improvements in alcohol knowledge and/or attitudes. In addition to these knowledge or attitude outcomes, the majority (62 %) of the CBIs showed significant reductions in alcohol related behaviors. The proportion of CBIs reporting significant behavioral outcomes was greater among those that used a broad theoretical framework (71 %) compared to those that targeted a specific theoretical construct and/or intervention technique (51 %).

**Table 3:** Description of studies and study outcomes for CBIs included in the literature review: studies of interventions which used a broad theory

Intervention name/Theories or constructs used	Author, year	Setting/Participants	Intervention description (including dose)	Comparator	Primary outcomes
21 Web BASICS • Theory of Planned Behavior	Neighbors C, 2009	295 university students intending to have 2 or more drinks on their 21st birthday	Single-sessions web-based personalized feedback sent with an electronic birthday card	• Assessment only control	• Estimated blood alcohol concentration on 21st birthday*
	Neighbors C, 2012	599 university students intending to binge drink on their 21st birthday	Single-session 21st Birthday Web –BASICS, personalized feedback covering intended drinking and drinking consequences	• 21st birthday in-person BASICS • 21st birthday in-person BASICS plus friend intervention • 21st birthday web BASICS plus friend intervention • BASICS • Attention control.	• Actual alcohol consumption • Actual estimated blood alcohol concentration * • Alcohol-related consequences during 21st birthday
Alcohol 101 • Social Cognitive Theory • Theory of Reasoned Action • Transtheoretical model	Barnett NP, 2004	117 mandated violators of college alcohol policy	Alcohol 101: Single 45-minute session featuring a virtual party	• Brief, in-person motivational intervention, no booster • Brief, in-person motivational intervention, plus booster session • Alcohol 101, plus booster session	• Frequency of drinking (number of days drinking and number of heavy drinking days in the past month) • Drinks per week
	Barnett NP, 2007	225 mandated violators of college alcohol policy	Alcohol 101: Single 45 min session	• One-on-one intervention delivered by counselors trained in motivational interviewing	Past month: • Number of drinking days [3, 12 months* (CBI inferior)] • Number of heavy drinking days [3, 12 months] • Average number of drinks per drinking day [3, 12 months* (CBI inferior)] • Average estimated BAC [3, 12 months] Past 90-days: • Help seeking [3,* (CBI inferior) 12 months*] • Alcohol problems [3, 12 months]
	Carey KB, 2009	198 mandated violators of college alcohol policy	Alcohol 101 Plus: 60 min single session	• Brief motivational intervention using personalized feedback, discussion of alcohol-related consequences	• Reductions in drinking [men, women* (BMI showed greater reductions)]
	Carey KB, 2010	677 mandated violators of college alcohol policy	Alcohol 101 Plus: 60 min single session	• In-person brief motivational intervention • Alcohol Edu for Sanctions • Delayed control	• Alcohol consumption* – females but not males reduced drinking more after the BMI than after either CBI • Alcohol problems • Recidivism
	Donahue B, 2004	113 undergraduates earning academic credit	Alcohol 101: Single 45-minute session	• 30 min of cognitive behavioral therapy	• Number of drinks consumed per occasion • Number of alcoholic drinks consumed* (favoring CBT) • Number of days drinking alcohol* (favoring CBT) • Awareness of the consequences of alcohol use* • Greater reported propensity to be cautious in situations involving alcohol*
	Lau-Barraco C, 2008	217 students who had at least 2 episodes of heavy drinking in the past month, drank between 5 and 40 drinks weekly, and had no history of alcohol treatment	Alcohol 101: 90 to 120 min	• Assessment-only control • Expectancy challenge (a 90–120 min exercise in which participants drink an unknown beverage and must guess who really drank alcohol)	• Number of standard drinks per week* (favoring the expectancy challenge) • Frequency of heavy episodic drinking* (favoring the expectancy challenge) • Alcohol Expectancy Questionnaire scores: global positive changes* (favoring the expectancy challenge), Social • Assertiveness sub-scale* (favoring the expectancy challenge), social and physical pleasure sub-scale, relaxation and tension reduction sub-scale, power & aggression sub-scale and sexual enhancement sub-scale
	Mastroleo NR, 2011	225 mandated violators of college alcohol policy	Alcohol 101 Plus: 60 min single session	• Brief, single-session intervention led by master’s or PhD level clinicians with or without a 25-min 1-month booster session • Alcohol 101 Plus and a 1-month 25-minutes booster session with the program	• Number of heavy drinking days [Alcohol 101 vs. brief counseling] • Average number of drinks per drinking day [Alcohol 101 vs. brief counseling] • Alcohol problems [Alcohol 101 vs. brief counseling]
	Murphy JG, 2010	74 college students recruited at a student health center	Alcohol 101 Plus: 90 min single session	• A single, face-to-face BASICS session	• Normative and self-ideal discrepancy* (favoring BASICS over Alcohol 101) • Motivation to change drinking* (favoring BASICS over Alcohol 101) • Total drinks per week • Past month frequency of heavy drinking
	Reis J, 2000	912 students 16–18 year old and 2,565 students 19–25 years old	Alcohol 101: preliminary version	• Assessment-only control (older and younger groups) • Alternative alcohol education program	• Expectations about the consequences of alcohol use (some measures*) • Self-efficacy to handle alcohol safely (some measures*) • Perceived peer norms regarding drinking [not reported]
	Sharmer L, 2001	370 undergraduates earning academic credit	Alcohol-101: 3 60-minute presentations in an interactive classroom setting	• Classrooms receiving teacher-centered motivational speech • Classrooms receiving assessment only	• Attitudes towards alcohol [4, 8,* 12 weeks] • Knowledge scores [4,* (control scored higher) 8,* (controls scored higher) 12 weeks] • Self-reported alcohol use behavior
AlcoholEdu • Expectancy theory • Social Cognitive Theory • Social Norms Theory	Croom K, 2009	3,216 incoming first-year college students	AlcoholEdu (2006 edition): An interactive 2- to 3-hour web-based alcohol prevention course presented in two parts	• Assessment only control	• Alcohol-related knowledge* • Likelihood of playing drinking games* • Likelihood of drinking alcohol • Number of drinks in past 2 weeks • Protective behaviors • Risk-related behaviors • High-risk drinking • Alcohol-related harms
	Hustad JTP, 2010	82 incoming first-year college students in fulfillment of a mandatory alcohol education requirement	AlcoholEdu and The Alcohol eCHECKUP TO GO	• Assessment only control	• Typical week alcohol consumption [eCHUG* and AlcoholEdu* vs. control] • Heavy episodic drinking [eCHUG* and AlcoholEdu* vs. control] • Typical and peak alcohol consumption [eCHUG* and AlcoholEdu* vs. control] • Alcohol-related consequences [AlcoholEdu* vs. control]
	Lovecchio CP, 2010	1,620 incoming first-year college students	AlcoholEdu, version 8.0	• Assessment only control	• Alcohol-related knowledge* • Total number of drinks consumed in past 2 weeks* • Heavy episodic drinking* • High risk alcohol behaviors • Protective alcohol behaviors • Responsible drinking behaviors (favoring control group)* • Negative drinking consequences: behavioral* and psychological • Acceptance of others’ alcohol use* and acceptance of others’ everyday alcohol use • Expectancies of alcohol use: positive* and negative;
	Paschall MJ, 2011	2,400 first-year college students at 30 universities	AlcoholEdu, version 9.0	• Assessment-only control	• Past-30-day alcohol use [Fall*, Spring] • Average number of drinks per occasion [Fall*, Spring] • Binge drinking [Fall*, Spring]
	Paschall MJ, 2011	Same as above	AlcoholEdu, version 9.0	• Assessment-only control	Reports of 7 types of alcohol-related problems: • Physiological [Fall*, Spring] • Academic [Fall, Spring] • Social [Fall*, Spring] • Driving under the influence/ riding with drinking drivers [Fall, Spring] • Aggression [Fall, Spring] • Sexual risk [Fall, Spring] • Victimization [Fall*, Spring] • All problems [Fall*, Spring]
	Wall A, 2006	3,552 members of fraternities and sororities at universities in the United States and Canada	Pre-2006 edition, version and duration not specified	• Assessment only control, post-test only	• Heavy drinking in past 2 weeks* • Negative academic consequences* • Negative physical health or work consequences • Drinking and driving* • Hangover/ mental impact* • Negative sexual consequences*
	Wall AF, 2007	20,150 college students, pre-enrollment, during enrollment, or in fulfillment of first-year requirement	AlcoholEdu (2006 edition)	• Delayed intervention control group	• academic consequences* • hangover/ mental impact* • heavy consumption days* • intentional risky behavior* • positive expectancies of alcohol use*
	Wyatt TM, 2013	14,310 first-year college students	AlcoholEdu (edition not specified)	• No control, quasi-experimental analysis of time-series data	• Substantial decreases in alcohol consumption (any consumption and heavy drinking) and alcohol- or drug-related negative consequences
Climate Schools: Alcohol Module/Alcohol and The CLIMATE Schools Combined • Social Influence Approach	Newton NC, 2009	764 13-year olds at ten secondary schools	Climate Schools: Alcohol and Cannabis prevention course (consisting of two sets of six 40 min lessons)	• Schools allocated to usual health classes	• Alcohol knowledge* • Alcohol consumption* • Alcohol expectancies • Alcohol-related harms
	Newton NC, 2009	764 13-year olds at ten secondary schools	Climate Schools: Alcohol (consisting of a set of six 40-minute lessons)	• Schools allocated to usual classes	• Alcohol knowledge [immediate,* 6-month follow-up*] • Alcohol use [immediate,* 6-month follow-up] • Alcohol expectancies [immediate, 6-month follow-up] • Frequency of drinking to excess [immediate, 6-month follow-up] • Alcohol-related harms [immediate, 6-month follow-up]
	Newton NC, 2010	764 13-year olds at ten secondary schools	Climate Schools: Alcohol (consisting of a set of six 40-minute lessons)	• Schools allocated to usual health classes	At 12-months: • Alcohol knowledge* • Average weekly alcohol consumption* • Frequency of drinking to excess* • Alcohol expectancies • Alcohol-related harms
	Vogl L, 2009	1,466 13-year-old, eighth-grade students	CLIMATE Schools: Alcohol (six lessons)	• Schools allocated to usual classes	• Alcohol knowledge* • Positive social expectancies of alcohol use* • Alcohol consumption [females,* males] • Alcohol-related harms [females,* males] • Frequency of binge drinking [females,* males]
College Alc • Problem Behavior Theory • Theory of Planned Behavior	Bersamin M, 2007	622 incoming first-year students	5-unit, 3-hour course including graphics and text, interactive animations, online assignments, readings, quizzes and video clips	• Assessment-only control	• Frequency of heavy drinking [baseline drinkers,* baseline non-drinkers] • Felt drunk [baseline drinkers,* baseline nondrinkers] • Alcohol-related consequences [baseline drinkers,* baseline non-drinkers]
	Paschall MJ 2006	370 incoming first-year students	Same as above	• Assessment-only control	At the end of the fall semester: • Alcohol-related knowledge* • Positive attitudes toward alcohol use* • Alcohol use • Heavy drinking • Alcohol-related problems • Alcohol expectancies (positive and negative) • Normative beliefs • Intentions to use harm-minimization approaches*
	Wyrick DL, 2005	65 college students, for academic credit	Same as above	Pre- vs. post-test design (no control)	• Normative alcohol beliefs* • Alcohol expectancies* • Alcohol-related attitudes • Heavy alcohol use • Problems associated with alcohol use*
Check Your Drinking • Social Norms Theory	Cunningham JA, 2012	425 college students meeting criteria for risky drinking	Check Your Drinking (University Edition) including national norms for age, gender and country of origin (US and Canada) and information on caloric content and impact on weight of alcohol	• Controls not provided access to Check Your Drinking	• AUDIT-C scores at 6-week follow-up* • 18 % of study participants randomized to receive the intervention reported using it
	Doumas DM, 2008	59 first-year student athletes in NCAA division 1	15 min Web-based program (an earlier version of Check Your Drinking)	• Online education (15 min on an educational Web page)	• Alcohol consumption [high risk drinkers,* low risk drinkers] • Perceptions of peer drinking [high risk drinkers,* low risk drinkers]
	Doumas DM, 2009	76 mandated violators of a university alcohol or drug policy	15 min Web-based program	• Alcohol module of The Judicial Educator	At 30-day follow-up: • Weekly drinking quantity* • Peak alcohol consumption* • Frequency of drinking to intoxication* • Estimates of peer drinking* • Alcohol-related problems
eCHECKUP TO GO (eCHUG) • Expectancy theory • Social Norms Theory	Alfonso J, 2013	173 mandated violators of college alcohol policy	A 10–15 min single session self-directed online module	Personalized feedback delivered face-to-face: • Individually • In groups	• Alcohol use (no between group differences) • Alcohol-related harms (no between group differences, significant reductions over time in CHUG group)
	Doumas DM, 2014	513 9th graders	eCHECKUP TO GO for high school students, 30-minute module	• A school that received assessment only	• Quantity of weekly drinking • Drinking frequency * • Alcohol-related consequences* • Positive alcohol expectancies* • Positive beliefs about alcohol* • Normative beliefs regarding peer drinking
	Doumas DM, 2009	80 first-year college students participating in a voluntary orientation seminar	A 10–15 min single session self-directed online module	• Assessment-only control	• Weekly drinking quantity [high risk students,* low risk students] • Frequency of drinking to intoxication [high risk students,* low risk students] • Alcohol-related problems [high risk students,* low risk students]
	Hustad JTP, 2010	See entry for this study under AlcoholEdu	--	--	--
	Murphy JG, 2010	207 college students enrolled in introductory courses reporting at least one past-month heavy drinking episode	eCHECKUP TO GO, used for approximately 40 min	• A single, face-to-face BASICS session • Assessment-only control	• Normative discrepancy • Self-ideal discrepancy * (favoring BASICS) • Motivation to change drinking • Total drinks per week* (favoring BASICS) • Past month frequency of heavy drinking* (favoring BASICS)
	Walters ST, 2007	106 first-year, heavy drinking college students	Standard eCHECKUP TO GO, duration not described	• Assessment-only control	Among those who reported at least one heavy drinking episode in the past month: • Drinks per week [8 weeks,* 16 weeks] • Peak blood alcohol level [8 weeks,* 16 weeks] • Alcohol-related consequences [8 weeks, 16 weeks] • Perceived drinking norms [8 weeks,* 16 weeks]
	Walters ST, 2009	279 college students who reported at least one heavy-drinking episode	Web-based personalized feedback modified from the electronic-Check-Up to Go	• a single motivational interviewing (MI) session without feedback • a single MI session with feedback • assessment only	• Drinks per week [MI with feedback significantly better than Web-based feedback at 3 and 6 months] • Peak blood alcohol content [MI with feedback significantly better than Web-based feedback at 3 and 6 months] • Alcohol-related problems [MI with feedback significantly better than Web-based feedback at 3 and 6 months]
Lifeskills Training CD-ROM • Social Learning Theory • Problem Behavior Theory • Self-derogation theory • Peer cluster theories	Williams C, 2005	123 sixth and seventh graders completing the program at home over summer break	10 sessions	• Assessment-only control	• Substance use frequency • Pro-drug attitudes* • Normative expectations for peer and adult substance use* • Anxiety reduction skills* • Relaxation skills knowledge*
Michigan Prevention and Alcohol Safety for Students (M-PASS) • Health Belief Model • Precaution Adoption Process Model • Theory of Planned Behavior • Transtheoretical Model	Bingham C, 2010	1,137 first-year college students	4 10- to 15-minute interactive online Sessions	• Assessment-only controls designated by dormitory	• Advanced stages of change* • Tolerance of drinking and drink/driving* • Reasons to drink reported* • Use of strategies to avoid at-risk drinking*
	Bingham C, 2011	Same as above	Same as above	Same as above	At 3-month follow-up: • Frequency/quantity of alcohol use* • Binge drinking* • Frequency of riding with a drink driver* • Using strategies to avoid high-risk drinking* • Frequency of drink-driving • Stages of change* • Tolerance of drinking • Reasons to drink* • Reasons not to drink* • Tolerance of drink driving*
PAS [Prevention of alcohol use in students] • Theory of planned behavior • Social cognitive theory	Koning IM, 2009	3,490 first-year high school students and their parents at school and school events	4 digital, classroom-based lessons plus a printed booster lesson a year later	• Parent intervention • Parent intervention combined with student CBI • Standard alcohol education curriculum	• Incidence of (heavy) weekly alcohol use [10 and 22 months] • Frequency of monthly drinking [10 and 22 months]
Project Fitness • Behavior-Image Model (which is supported by Prospect Theory)	Moore MJ, 2012	200 students approached in a university’s common areas	Single 20-minute session on 7 health behaviors including alcohol use, that asks screening questions and provides gain-framed messages about healthy choices	• Assessment-only control	Immediately following intervention: • Alcohol intentions* • Alcohol prototype image [perceived similarity to those who drink]* • Willingness to be seen as someone who drinks a lot* • Alcohol behavior coupling [whether alcohol is perceived to interfere with other health behaviors] • Alcohol social norms*
Reach Out Central • Elaboration likelihood model • Social Cognitive Theory	Shandley K, 2010	266 18–25 year olds playing independently, recruited through online advertisements or invitations from secondary school teachers and university lecturers	An open-ended web-based interactive game in which a character explores and interacts with a virtual environment, no set length	• Pre-, post-evaluation with 2-month follow-up	• Alcohol use [females*, males] • Use of coping strategies [females*, males] • Psychological distress [females*, males] • Resilience and satisfaction with life [females*, males] • Mental health literacy [females*, males*] • Help-seeking [females*, males*]
RealTeen • Social Learning Theory	Schwinn TM, 2010	236 13- and 14-year-old girls recruited through a youth-oriented web site	A homepage (offering features accessible at any time) and 12 intervention sessions taking about 25-minutes each	• Assessment-only control	• Alcohol use [post-test, 6-month follow-up*] • Marijuana use [post-test, 6-month follow-up*] • Poly drug use [post-test, 6-month follow-up*] • Total substance use (alcohol and drugs) [post-test, 6-month follow-up*]
What Do You Drink • I-change Model (integration of several approaches including Fishbein-Ajzen’s Theory of Reasoned Action, TTM and Social Learning Theory) • Social Cognitive Theory	Voogt CV, 2013	907 18- to 24-year olds reporting heavy drinking in the past 6 months and motivation to change their alcohol use	A brief online intervention including personalized normative feedback, a segment in which participants set a goal for their drinking, and a portion on refusal strategies	• Assessment-only control	• Weekly alcohol consumption [1 month, 6 months] • Frequency of binge drinking [1 month, 6 months] • Heavy drinking [1 month, 6 months]
	Voogt CV, 2014	Same as above	Same as above	• Assessment-only control	• Drinking refusal self-efficacy*
Your Decisions Count– Alcohol, Tobacco and Other Drugs • Transtheoretical Model	Evers KE, 2012	1,590 students in grades 6–9 who reported having ever using alcohol, tobacco, marijuana, or other drugs	Three 30-minutes internet-based modules	• Assessment-only control	• Percentage of “ever-users” who were using alcohol, tobacco, marijuana, and other drugs [3 months,* 14 months] • Likelihood of moving into action/maintenance stage of change [3 months,* 14 months] • Post-test Cessation Rates Among current substance users [3 months,* 14 months]
No name [Asian-American Mother Daughter Intervention] • Family interaction theory	Fang L, 2010	108 Asian–American girls aged 10–14 years and their mothers recruited online or through community service agencies	9-session web-based substance use prevention program with each session taking about 45 min	• Assessment-only control	1-year follow-up: • Depressed mood* • Self-efficacy and refusal skills* • Levels of mother–daughter closeness* • Mother–daughter communication* • Maternal monitoring * • Family rules against substance use* • Instances of alcohol, marijuana, and illicit prescription drug use* • Intentions to use substances in the future*
	Fang L, 2013	Same as above	Same as above	• Assessment-only control	2-year follow-up: • Depressed mood • Self-efficacy and refusal skills* • Levels of mother–daughter closeness* • Mother–daughter communication* • Maternal monitoring * • Family rules against substance use* • Instances of alcohol, marijuana, and illicit prescription drug use* • Intentions to use substances in the future* • Substance use normative beliefs • Body esteem
No name [Black and hispanic mother-daughter intervention] • Attachment Theory • Deviant behavior proneness theory • Family interaction theory • Social Learning Theory	Schinke S, 2011	546 pairs of girls ages 10 to 13 and their mothers from New York, New Jersey, and Connecticut recruited from postings on craigslist.org and advertisements in New York City newspapers	10 sessions with varying completion times amongst the participants	• Assessment-only control	• Mother-daughter communication [reported by daughter,* reported by mother] • Perceptions of family rules against substance use [reported by daughter,* reported by mother] • Perceptions of parental monitoring of extracurricular activities, whereabouts, and friends [reported by daughter,* reported by mother] • Daughters’ normative beliefs about peer substance use* • Depression among daughters* • Self-efficacy to avoid alcohol, tobacco and drug use among daughters * • Alcohol use among daughters * • Daughters’ intentions to smoke, drink, and use drugs when they are adults*
No name [College freshman intervention] • Social Comparison Theory • Social Identity Theory • Social Impact Theory	Lewis MA, 2007	316 college students in psychology classes who indicated at least one heavy drinking episode	After a baseline survey, gender-specific or gender-neutral personalized feedback provided on screen and as a print-out	• Assessment-only control	• Overall alcohol consumption* • Average number of drinks consumed/past month • Typical number of drinks consumed/occasion* • Typical drinking frequency*
	Lewis MA, 2007	185 first-year college students reporting at least one heavy-drinking episode in the past month	Same as above	• Assessment-only control	• Perceived same-sex norms surrounding drinking behavior [gender-specific PNF*, gender-neutral PNF] • Perceived gender-neutral norms surrounding drinking behavior [gender-specific PNF*, gender-neutral PNF*] • Drinks per week [gender-specific PNF,* gender-neutral PNF] • Drinking frequency [gender-specific PNF*, gender-neutral PNF*]
No name [E-newsletter intervention] • Extended Parallel Process Model (based on Social Cognitive Theory and the Health Belief Model)	Moore MJ, 2005	116 juniors and seniors enrolled in 3 college courses aged 18 to 25 years with access to an active e-mail account	A series of 4 weekly newsletters in electronic format	• Newsletters in print format	• Past-year drinking frequency • Past 30-day drinking frequency • Quantity • Binge-drinking frequency • Get “drunk” frequency • Get “drunk” quantity • Greatest number of drinks • 2-week binge-drinking frequency
No name [Laptop ER intervention] • Social Learning Theory	Gregor MA, 2003	671 patients aged 14 to 18 years presenting to the ED within 24 h after an acute minor in- jury	Single-session approximately 25 min long	• None	• Attitudes about their alcohol use*
	Maio RF, 2005	Same as above	Same as above	• Assessment-only control	• Alcohol Misuse Index scores [3 months, 12 months] • Binge-drinking episodes [3 months, 12 months]
No name [Web-based Substance Use Prevention for Adolescent Girls] • Family interaction theory • Self-efficacy • Manipulation subjective social norms • Cognitive behavioral therapy	Schinke S, 2009	202 girls ages 10 to 13 and their mothers from New York, New Jersey, and Connecticut recruited through online or print advertising	14 computer-mediated intervention modules (duration not reported)	• Assessment-only control	At two-month follow-up: • Alcohol consumption in the past 7 days,* 30 days,* and year* • Conflict management and alcohol-use refusal skills* • Mother-daughter communication skills* • Daughters’ report of parental monitoring and rule setting* • Normative beliefs about underage drinking* • Self-efficacy about their ability to avoid underage drinking* • Intentions to drink as adults* • Mother-daughter communication skills [reported by daughters*, reported by mothers*] • Parental monitoring and rule setting [reported by daughters,* reported by mothers*]
	Schinke S, 2009	916 girls 11 to 13 and their mothers from New York, New Jersey recruited through radio, print, internet and public transit advertising	9 computer-mediated intervention modules, each taking approximately 45 min	• Assessment-only control	At two-year follow-up: • Alcohol consumption in the past 30 days [immediate follow-up, 1 year follow-up*]
	Schinke S, 2009	591 girls 11 to 13 and their mothers from New York, New Jersey recruited through radio, print, internet and public transit advertising	Same as above	• Assessment-only control	At one-year follow-up: • Alcohol consumption in the past 30 days [2 year follow-up*]

Asterisk indicates intervention outcomes for which statistically significant inter-group differences were found

**Table 4:** Description of studies and study outcomes for CBIs included in the literature review: studies of interventions which did not use a broad theory

Intervention name	Author, year	Setting/ Participants	Intervention description (including dose)	Comparator	Primary outcomes
AMADEUS Manipulating subjective social norms	Ekman DS, 2011	654 third-semester university students	Personalized normative feedback consisting of 12 possible statements or suggestions about the student's alcohol use	• Control receiving very brief feedback consisting of three statements	• Average weekly alcohol consumption [3 months, 6 months] • Proportion with risky alcohol consumption [3 months, 6 months] • Frequency of heavy episodic drinking [3 months, 6 months] • Peak blood alcohol concentration [3 months, 6 months]
	McCambridge J, 2013	14,910 students in semesters 1, 3 and 5 of their studies during the autumn term at two Swedish universities	A 10-item alcohol assessment with personalized normative feedback comparing users’ alcohol use to peers and offering advice on the importance of limiting unhealthy drinking	• Alcohol assessment only without feedback • No contact (neither assessment nor feedback)	• Prevalence of risky drinking [alcohol assessment without feedback, no contact*] • AUDIT-C scores [alcohol assessment without feedback, no contact]
College Drinker’s Check-up • Manipulating subjective social norms	Hester RK, 2012	144 (study 1) and 82 (study 2) college student volunteers 18–24 who met criteria for heavy, episodic drinking	Screening followed by 3 modules which took ~35 min, including decisional balance exercises, assessment of risks associated with alcohol use, and personalized normative feedback,	• Assessment-only control • Delayed-assessment control	• Standard Drinks per Week (1 month,* 12 months* • Peak BAC in a Typical Week (1 month,* 12 months) • Average Number of Drinks during two heaviest episodes in the past month (1 month,* 12 months*) • Average Peak BAC during two heaviest episodes in the past month (1 month,* 12 months *)
Drinker’s Assessment and Feedback Tool for College Students (DrAFT-CS) • Motivatoinal interviewing • Social norms theory	Weaver CC, 2014	176 heavy drinking college students recruited from undergraduate psychology courses	45-minute, single-session personalized feedback session	• DrAFT-CS plus moderation skills (DrAFT-CS+) • Moderation skills only • Assessment only	• Estimated blood alcohol concentrations on typical heaviest drinking day (DrAFT-CS and DrAFT-CS+ vs. assessment-only group*) • Drinks per week (DrAFT-CS+ vs. assessment-only group,* all other comparisons non-significant) • Peak drinking episode (DrAFT-CS+ vs. assessment-only group,* all other comparisons non-significant)
e-SBINZ • Manipulating subjective social norms	Kypri K, 2013	1,789 Maori university students who screened positive for hazardous or harmful drinking	Single session of web-based alcohol assessment and personalized feedback taking less than 10 min	• Assessment-only control	• Drinking frequency * • Drinks per occasion* • Total volume of alcohol consumed, past 28 days* • Academic problems associated with alcohol use*
Head On, for grades 6 through 8 • Manipulating subjective social norms	Marsch LA, 2007	272 students in grades 6 through 8	15 sessions throughout the school year	• 15 sessions of in-person Life Skills Training	• Knowledge related to substance use prevention* • Self-reported alcohol use • Intentions to use substances • Attitudes towards substances • Beliefs about prevalence of substance use among peers and adults
iHealth • Manipulating subjective social norms • Motivational interviewing • Self-change approaches	Saitz R, 2007	4,008 first-year college students recruited through an email invitation	The minimal intervention [see comparator condition] plus 3 screens providing feedback about personal consequences, costs, and caloric content of user’s alcohol use	• Minimal online brief intervention: an online module consisting of 3 screens of personalized normative feedback	• Readiness to change [women,* men] • Proportion willing to seek help for unhealthy alcohol use [women, men*] • Percentage of participants no longer reporting unhealthy alcohol use one month later • Drinks per week • Drinks per occasion
In Focus • Manipulating subjective social norms	Gare L, 1999	1,000 students ages 12 and 13	4 lessons each lasting approximately 40 min	• Assessment-only controls	• Substance use knowledge* (but no change observed on alcohol-specific questions) • Substance use attitudes • Substance use intentions
MyStudentBody.com • Manipulating subjective social norms	Chiauzzi E, 2005	265 students at five public and private, 2-year and 4-year colleges	Four weekly 20-minute sessions	• Alcohol education web site as control	• Binge drinking days/week • Maximum number of drinks/drinking day, past week* • Quantity of consumption • Frequency of consumption • Average consumption • Alcohol composite score* • Peak consumption during special occasions [women*, men] • Total consumption during special occasions [women*, men] • Alcohol related problem behavior [women*, men] • Readiness to change
Project Chill • Motivational interviewing • Manipulating social norms • Self-efficacy	Walton MA, 2013	328 12–18 year-olds at community health clinics reporting past-year cannabis use	Single-session stand-alone interactive animated program	• Assessment-only control • Therapist based intervention	• Cannabis use [3 months, 6 months, 12 months] • Cannabis related consequences [3 months*, 6 months, 12 months] • Alcohol use [3 months, 6 months, 12 months] • Driving under the influence [3 months, 6 months, 12 months]
	Walton MA, 2014	714 12–18 year-olds at community health clinics reporting no lifetime cannabis use	Single-session stand-alone interactive animated program (average duration of 33 min)	• Assessment-only control • Therapist based intervention	• Any cannabis use [3 months, 6 months, 12 months*] • Frequency of cannabis use [3 months*, 6 months*, 12 months] • Frequency of other drug use [3 months*, 6 months, 12 months] • Severity of alcohol use [3 months, 6 months, 12 months]
Refusal Challenges • Self-efficacy	Bryson R, 1999	180 8th-grade students (primarily Hispanic) in rural Southern California	Program played in pairs for one hour a day, typically finished in two days	Assessment-only control	• Refusal skill scores [posttest*, follow-up*]
SafERteens • Motivational interviewing • Social norms theory • Social Cognitive Theory • Transtheoretical Model • Theory of planned behavior • Health belief model	Cunningham RM, 2009	533 patients ages 14 to 18 who presented to the emergency department for illness or injury and reporting past-year violence and alcohol use	35-minute single session interactive, animated program including tailored feedback, exercises identifying reasons to stay away from drinking and fighting, and role-play scenarios	• Assessment-only control • Therapist-delivered intervention	Relative to assessment-only control: • Alcohol use [post-test, 3 month follow-up] • Attitudes toward alcohol and violence [post-test*, 3 month follow-up*] • Self-efficacy for avoiding alcohol [post-test*, 3 month follow-up] • Readiness to change alcohol use [post-test, 3 month follow-up]
	Cunningham RM, 2012	726 patients ages 14 to 18 who presented to the emergency department for illness or injury and reporting past-year violence and alcohol use	Same as above (median time to complete was 29 min)	• Assessment-only control • Therapist assisted by a computer	• Peer aggression [computer, therapist*] • Peer victimization [computer, therapist*] • Violence-related consequences [computer, therapist] • Alcohol misuse [computer, therapist] • Binge drinking [computer, therapist] • Alcohol-related consequences [computer, therapist]
THRIVE (Tertiary Health Research Intervention Via Email) • Manipulating subjective social norms	Kypri K, 2009	2,435 undergraduates reporting unhealthy drinking	Age- and gender-specific personalized feedback including explanation of the user’s AUDIT score, the calories in and costs of drinking, and links to other resources	• Assessment-only control	• Drinking frequency [1 month*, 6 months*] • Typical occasion quantity of alcohol consumed [1 month*, 6 months] • Overall volume of alcohol consumed [1 month*, 6 months*] • Personal and academic problems score [1 month, 6 months] • Prevalence of binge drinking [1 month, 6 months] • Prevalence of heavy drinking [1 month,* 6 months*]
No name [At-risk university students personalized normative feedback] • Manipulating subjective social norms	Butler LH, 2009	84 undergraduates who reported at least two binge episodes and two alcohol related problems in the past 28 days	A single session in which participants spent an average of 11 min reviewing their feedback	• Assessment-only control • Face-to-face intervention	• Drinks per week [CBI vs. face-to-face, CBI vs. control*] • Drinking occasions per week [CBI vs. face-to-face, CBI vs. control*] • Binge drinking days/month [CBI vs. face-to-face*, CBI vs. control] • Rutgers Alcohol Problem Index scores [CBI vs. face-to-face, CBI vs. control]
No name [Blood Alcohol Concentration Feedback] • Personalized normative feedback	Thombs DL, 2007	386 residents of certain freshman dormitories, once a night, Wednesday. through Saturday	Residents’ blood alcohol concentration assessed at night. Readings and normative feedback available online the next day	• Students in dormitories in which blood alcohol level but not information on norms was reported	• Observed blood alcohol content* (lower in comparator group)
No name [E-mailed personalized normative feedback for college students] • Manipulating subjective social norms	Bryant ZE, 2013	310 college students enrolled in introduction to psychology courses	A single e-mail containing personalized feedback on alcohol use	• E-mailed generic feedback	• Drinks in a given week* • Number of days being drunk in the previous 30 days* • Number of days they perceived their peers to have drunk alcohol* • Amount of alcohol they perceived their peers to have consumed per drinking occasion*
No name [Gender-specific personalized feedback to reduce alcohol use among college Students] • Social Comparison Theory • Social Identity Theory • Self-categorization Theory	Neighbors C, 2010	818 first-year college students who engaged in binge drinking at least once in the past month	“Extremely brief” gender-specific and gender-nonspecific personalized normative feedback based on a 50-minute survey delivered a single time or biannually	• Attentional control	• Typical weekly drinking amount • Alcohol-related problems • Heavy episodic drinking
No name [Intervention to reduce alcohol use among hazardous drinking college Students] • Personalized normative feedback	Palfai TP, 2011	119 hazardous drinking students in an introduction to psychology class	Single-session gender and university-specific personalized normative feedback on alcohol consumption and drinking consequences, plus information on costs and calories associated with drinking	• Information on healthy eating and sleep habits	• Number of drinks per week* • Episodes of heavy drinking
No name [New Zealand university student presonalized normative feedback] • Manipulating subjective social norms	Kypri K, 2004	104 students recruited in reception area of the student health service who screened positive on an AUDIT test	10–15 min of web-based assessment and personalized feedback	• Assessment-only control	• Total alcohol consumption [6 weeks,* 6 months] • Heavy drinking episode frequency [6 weeks,* 6 months] • Number of personal problems [6 weeks,* 6 months*] • Academic problems score [6 weeks, 6 months*]
	Kypri K, 2008	576 students attending a university health care service who screened positive for hazardous drinking	Personalized feedback, delivered either once or 3 times (1 and 6 months after the intervention)	• Informational pamphlet	• AUDIT scores [12 months: single-dose,* multi-dose*] • Frequency of drinking [6 months: single-dose,* multi-dose,* 12 months: single-dose, multi-dose] • Typical drinking occasion quantity [6 months: single-dose, multi-dose, 12 months: single-dose, multi-dose] • Total alcohol consumption [6 months: single-dose,* multi-dose,* 12 months: single-dose,* multi-dose] • Very heavy drinking episode frequency [6 months: single-dose, multi-dose,* 12 months: single-dose, multi-dose] • Number of personal problems [6 months: single-dose, multi-dose, 12 months: single-dose, multi-dose] • Academic problems score [6 months: single-dose,* multi-dose,* 12 months: single-dose,* multi-dose*]
No name [Primary care intervention for multiple health risk behaviors] • Personalized normative feedback	Kypri K, 2005	218 university students 17–24 attending a student health service	Feedback on reported health behaviors with information on official guidelines and norms among peers	• Assessment-only control • Minimal contact (at baseline blood pressure and demographics but no assessment of behaviors)	• Prevalence of hazardous drinking • Peak estimated blood alcohol concentration
No name [U.K. college student personalized normative feedback] • Manipulating subjective social norms	Bewick BM, 2008	506 respondents to a university-wide student survey	Online personalized feedback with sections on levels of alcohol consumption, social norms, and standard advice and drinking information	• Assessment-only control	• CAGE score • Average number of alcoholic drinks consumed per drinking occasion* • Alcohol consumption over the last week
No name [Intervention to change sexual and alcohol norms for college students] • Personalized normative feedback	Patrick ME, 2014	271 college students between the ages of 18 and 21 who planned to go on a spring break trip with their friends	Personalized feedback intervention covering drinking and sex over spring break, reasons to avoid risky alcohol use, and behavior pacts with friends	• Assessment-only control	• Maximum drinks reported over spring break • Total drinks reported over spring break • Perceived norms for spring break drinking and sex* • Protective behavioral strategies • Spring break sexual behavior • Alcohol-related consequences reported over spring break • Sex-related consequences over spring break

Asterisk indicates intervention outcomes for which statistically significant inter-group differences were found

## Discussion

This study identified 100 unique articles covering 42 unique computer-based interventions (CBIs) aimed at preventing or reducing alcohol use among adolescents and young adults. Half of these CBIs have not been included in previous reviews. Thus, this review includes a total of 21 new CBIs and 43 new articles.

This review is the first to provide an in-depth examination of how CBI’s integrate theories of behavior change to address alcohol use among adolescents and young adults. While theories of behavior change are a critical component of effective interventions that have been developed and evaluated over the past several decades [51, 52], attention to the application of theory in CBIs has been limited. We utilized a simple classification system to examine if theories were mentioned, applied or measured in any of the publications that corresponded with the CBIs.

Only half of the CBIs reviewed mentioned use of an overarching, established theory of behavior change. The other half mentioned used of a single construct and/or intervention technique but did not state use of a broader theory. CBIs that were based on a broad theoretical framework were more likely to include measures of constructs associated with the theory than those that used a discrete construct or intervention technique. However, greater attention to what theory was used, articulating how theory informed the intervention and including measures of the theoretical constructs is critical to assess and understand the causal pathways between intervention components/mechanisms and behavioral outcomes (that would be predicted according to the theory). When mentioning the use of a theory or construct, almost all provided at least some description of how it informed the CBI; however, the amount and quality of information about how the theory was applied to the intervention varied considerably. Greater attention to what is inside the “black box” is critical in order to improve our understanding of not only what works, but why it works. While a few articles provided detailed information about the application of theory, the majority included limited information to examine the pathway between intervention approach and outcomes.

There are a number of reasons why there may be limited information on the use of theory in CBIs. Some researchers/intervention developers may not fully appreciate how theory can be used to inform intervention approaches. There is an emphasis on outcomes/effectiveness of interventions and less attention is placed on their development. In addition, to our knowledge, there are no publication guidelines/standards for describing the use of theoretical frameworks in intervention studies and the inclusion of this information is often up to individual authors and reviewers. Given the importance of theory in guiding interventions, greater emphasis on the selection and application of theory is needed in publications. The classification system used in this review (and originally developed by Painter [22], can serve as a simple framework for intervention developers, authors and journal reviewers so that there is greater consistency in the information provided on how theories are mentioned applied and measured in CBIs.

While there was considerable variation in how theory or constructs were applied to the CBIs, almost all (26) provided some form of personalized normative feedback and applied it relatively consistently across the CBIs. Personalized normative feedback is designed to correct misperceptions about the frequency and acceptability of alcohol use among peers. It typically involves an assessment of a youth’s perceptions of peer norms around alcohol attitudes and use followed by tailored information about actual norms [53]. In addition, some interventions have recently incorporated personal feedback to address individual’s motivations to change through assessing and providing feedback on drinking motives [54] or in decisional balance exercises [55]. The widespread use of personalized normative feedback in CBIs may be because it has been widely documented as an effective strategy and because it lends itself readily to an interactive, personalized computer-based intervention. Motivational interviewing was also used in several of the CBIs and is an effective face-to-face counseling technique [56]. In contrast, this technique was applied to CBIs in a number of different ways, such as exercises designed to clarify goals and values, making both the description of how it was applied even more essential to examine differential effectiveness across various CBIs.

This study builds on the growing evidence supporting the use of CBIs as a promising intervention approach. We found most of the CBIs improved knowledge, attitudes and reduced alcohol use among adolescents and young adults. In addition, this study suggests CBIs that use overarching theories more frequently reported significant behavioral outcomes than those that use just one specific construct or intervention technique (in isolation from a broader theory). This finding is consistent with prior studies examining the use of theory in face-to-face interventions targeting alcohol use in adolescents [57]. However, it is important to acknowledge the wide variation across the CBIs not only in their use of theory, but in scope, the targeted populations, duration/dosage, and measured outcomes. It is encouraging that even brief/targeted CBIs demonstrated some effectiveness and thus can play an important role in improving knowledge and attitudes, which are important contributors to changes in behavior.

There are limitations to this study. As discussed previously, many articles did not explicitly describe how theory was applied in the CBI. It is therefore possible that the theoretical pathways for the intervention were further developed than we have noted, and possibly included in other documents, such as logic models and/or funding applications; however, such information is not readily accessible and was outside the scope of this review. Thus, lack of mention of the name of a theory or construct or its application does not mean that the intervention did not integrate the theory in the intervention, only that the article did not provide information about its application. Thus, due to variations in the described use of theory along with the wide range of CBIs, it was not possible to draw comparisons about the relative effectiveness of CBIs according to the theory used. The ability to make such comparisons is further limited by the wide time frame in which CBIs were developed. This review spanned articles published between 1995 and 2014. During this period, CBIs to address health issues have been rapidly evolving due to major advancements in technological innovations (e.g., touch screen capabilities, mobile computing, improved graphics and user interfaces, and adaptive interface technologies features, etc.). These advancements coupled with greater interest and investments from federal agencies and philanthropic foundations. Over time one would expect these factors to further contribute to the effectiveness of CBIs.

## Conclusion

This study points to the promise of CBIs for reducing alcohol use, as well as gaps in the use and application of theory in the development and testing of these interventions. This study provides a useful framework for articulating explanatory pathways leading to behavioral outcomes. Unlike traditional curriculum-based, face-to-face interventions, CBIs offer a great deal of flexibility with regards to when and where they can be delivered. Across the 42 CBIs in this study, some (33) were delivered in schools, but many were used at home or in a clinic setting. However, CBIs are often stand-alone interventions that have not been integrated into broader intervention delivery systems (e.g., schools or health care systems), potentially limiting the impact of the CBI. Future research should explore how CBIs can be integrated into broader intervention efforts that take place in schools, clinics, and other community-based settings, while ensuring the privacy and confidentiality of adolescents’ sensitive health [58].

## Footnotes

1 Defined as consuming four or more alcoholic drinks per occasion for women and five or more for men [59].
